# Predictive performance of NEWS and qSOFA in immunocompromised sepsis patients at the emergency department

**DOI:** 10.1007/s15010-024-02247-4

**Published:** 2024-04-12

**Authors:** Lisanne Boekhoud, Helena M. E. A. Schaap, Rick L. Huizinga, Tycho J. Olgers, Jan C. ter Maaten, Douwe F. Postma, Hjalmar R. Bouma

**Affiliations:** 1 Department of Clinical Pharmacy and Pharmacology, University Medical Center Groningen, University of Groningen, P.O. Box 30.001, EB70, 9700 RB Groningen, The Netherlands; 2 Department of Internal Medicine, University Medical Center Groningen, University of Groningen, Groningen, The Netherlands

**Keywords:** Sepsis, Immunocompromised, Early recognition, Emergency department, (q)SOFA, NEWS

## Abstract

**Purpose:**

Sepsis has a high incidence and a poor prognosis. Early recognition is important to facilitate timely initiation of adequate care. Sepsis screening tools, such as the (quick) Sequential Organ Failure Assessment ((q)SOFA) and National Early Warning Score (NEWS), could help recognize sepsis. These tools have been validated in a general immunocompetent population, while their performance in immunocompromised patients, who are particularly at risk of sepsis development, remains unknown.

**Methods:**

This study is a post hoc analysis of a prospective observational study performed at the emergency department. Inclusion criteria were age ≥ 18 years with a suspected infection, while ≥ two qSOFA and/or SOFA criteria were used to classify patients as having suspected sepsis. The primary outcome was in-hospital mortality.

**Results:**

1516 patients, of which 40.5% used one or more immunosuppressives, were included. NEWS had a higher prognostic accuracy as compared to qSOFA for predicting poor outcome among immunocompromised sepsis patients. Of all tested immunosuppressives, high-dose glucocorticoid therapy was associated with a threefold increased risk of both in-hospital and 28-day mortality.

**Conclusion:**

In contrast to NEWS, qSOFA underestimates the risk of adverse outcome in patients using high-dose glucocorticoids. As a clinical consequence, to adequately assess the severity of illness among immunocompromised patients, health care professionals should best use the NEWS.

**Supplementary Information:**

The online version contains supplementary material available at 10.1007/s15010-024-02247-4.

## Introduction

Sepsis is a life-threatening syndrome of organ dysfunction caused by a dysregulated host response to infection that affects 50 million patients worldwide annually [1]. Despite efforts to improve recognition and treatment, sepsis remains an important cause of morbidity and mortality among patients admitted to the hospital with mortality rates ranging from 20 to 36% [2–7]. Timely initiation of adequate treatment is essential to prevent clinical deterioration. Therefore, the key to reducing sepsis-related mortality could lie in improving early recognition and identification of those at risk of poor outcomes [7, 8]. Currently, the most commonly used clinical assessment tools to facilitate early recognition of sepsis are the Systemic Inflammatory Response Syndrome (SIRS, Sepsis-2) criteria, (quick) Sequential Organ Failure Assessment ((q)SOFA, Sepsis-3), and the National Early Warning Score (NEWS) [1]. Of these scores, the NEWS and SOFA appears to have the highest accuracy to predict sepsis-related mortality [9–11]. Although the NEWS was not specifically developed for early sepsis recognition, it can accurately identify patients with more severe disease and thereby facilitate early recognition of sepsis [9, 10].

Most research validating qSOFA and NEWS are performed among general emergency department (ED) and ICU patients—not explicitly assessing the scores in immunocompromised patients. Yet, immunocompromised patients with an infection are at increased risk for sepsis development. Early recognition of sepsis and risk prediction in immunocompromised patients might be more difficult due to their altered immune response. Data describing the prognostic accuracy of the qSOFA and NEWS in immunocompromised patients are scarce and are either contradicting or cannot be generalized to pharmacologically immunocompromised patients. Immunodeficiency due to co-morbidity (i.e., cancer, neutropenia, AIDS) is a risk for 28-day mortality among patients with sepsis admitted to the ICU [2]. Unexpectedly, patients with bacteremia after solid organ transplantation have a lower 28-day and 90-day mortality as compared to age-, sex-, and hospital-matched bacteremia patients without history of organ transplantation [12]. The performance of SIRS, qSOFA, and NEWS has been assessed in patients with a suspected infection after hematopoietic cell transplantation. In this population, the NEWS had a moderate sensitivity (78%) and specificity (70%), which outperformed qSOFA and SIRS [13]. Together, immunosuppression due to co-morbidity affects outcome in patients with sepsis, with relevance to the predictive performance of commonly used sepsis scores.

The effect of immunosuppressive medication on clinical outcomes in patients with sepsis, and thereby, the predictive performance of sepsis scores remains unknown. We hypothesized that dampening the immune response by immunosuppressive drugs affects the prognostic accuracy of NEWS and qSOFA, which is likely to depend on drug class. For this study we used the NEWS in addition to the qSOFA, as advised by the Dutch sepsis guideline to support sepsis recognition at the ED [14]. To this end, we compared the prognostic performance of NEWS and qSOFA to predict the in-hospital mortality in pharmacologically immunocompromised patients with sepsis at the ED, adjusted to the immunosuppressive drug class.

## Methods

### Study design

This is a post hoc analysis of a prospective observational cohort study of adult patients visiting the ED of the University Medical Center Groningen (UMCG), a tertiary medical center. This study adhered to the Strengthening the Reporting of Observational Studies in Epidemiology (STROBE) and Transparent Reporting of a multivariable prediction model for Individual Prognosis or Diagnosis (TRIPOD) criteria recommendation for cohort studies [15, 16]. The Dutch Medical Research Involving Human Subjects Act is not applicable for this study, as ruled by the Institutional Review Board of the University Medical Center Groningen, and a waiver was granted (METc 2015/164). Written informed consent was obtained from all patients included in this study.

### Population

**Fig. 1:**
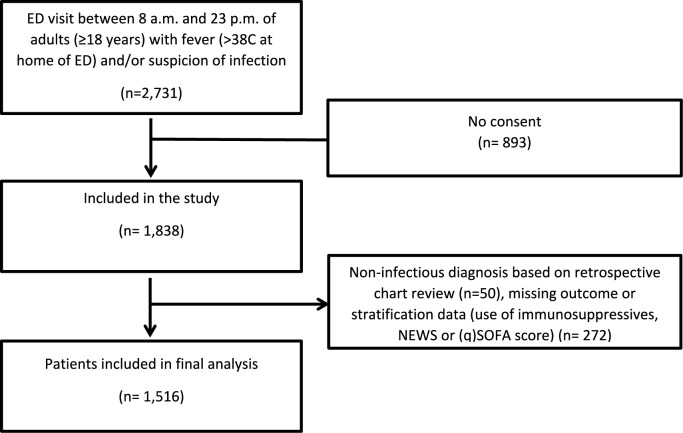
Flow chart of patient selection. Adult medical patients visiting the ED of the UMCG between March 2016 and July 2020 were screened for inclusion

From March 2016 to July 2020, 39,719 adult patients (≥ 18 years of age) visiting our ED for internal medicine, gastroenterology, rheumatology, emergency medicine (non-trauma), or pulmonology, of which 31,331 visits occurred between 8:00 and 23:00 h, were screened for inclusion. Patients were only screened between 8:00 and 23:00 due to the availability of the research team. Patients who presented with a suspected infection (as determined by the treating physician upon initial contact based on focal symptoms suggestive of an infection [e.g., productive cough, dyspnea, dysuria, pollakiuria, abdominal pain, erythema]) and/or fever (≥ 38 °C, either at home or upon triage in the ED) were included by a trained team of researchers. This approach allowed to include data from a heterogeneous, real-world population of ED visitors with a clinically relevant infection, including patients with sepsis. Patients with an alternative non-infectious diagnosis upon ED visit (*n* = 50) were excluded, as were patients with missing outcome or stratification data (use of immunosuppressives, NEWS or (q)SOFA score) (*n* = 272). Thereby, 1516 subjects were included in the dataset for the current analysis (Fig. 1).

### Data collection

Collected data included demographic characteristics, vital parameters, and laboratory measurements at admission. Patient characteristics consisted of age, sex, history of diabetes mellitus, chronic kidney disease, kidney transplantation, cardiovascular disease (defined as chronic heart failure and/or ischemic heart disease), and active cancer (defined as radiotherapy or chemotherapy treatment received up to 2 years prior to the current hospitalization). Data were collected from the electronic patient files, by shadowing and observing, as well as by interviewing patients and physicians. Data about treatment which was given outside the ED had not been collected. Information regarding use of immunosuppressive agents was subtracted from the patient’s medical record. The dosage of glucocorticoids was transformed into the equivalent prednisone dosage, based on the defined daily dose (DDD) as published by the World Health Organization (WHO) Collaborating Centre for Drug Statistics Methodology.

### Outcomes

The primary outcome was in-hospital mortality, while secondary outcomes were ICU admission and 28-day mortality. Patients were allocated to the immunosuppressives group when using immunosuppressive drugs at the time of presentation to the ED [17]. Table S1 in the appendix provides an overview of the drugs included in each class. The relative high number of patients using glucocorticoids allowed us to subdivide the groups based on the dosage, being < 7.5 mg/day (low dose), 7.5–15 mg/day (intermediate dose), and ≥ 15 mg/day (high dose).

### Statistical analysis

Statistical analyses were performed using R studio version 3.5.1 (RStudio, Inc., Boston, MA, USA). The following packaging were used: haven, lattice, ggplot2, ggpubr, ggsignif, tableone, cowplot, readxl, and GGally. Differences between two groups were compared using a two-tailed independent sample *t*-test, if normally distributed. In case of non-normally distributed data, a Mann–Whitney *U* test was used. Categorical variables were compared using a Chi-square test. In all cases, *p* < 0.05 was considered as a significant difference. Multiple univariate regression analyses were used to explore the association between the class of immunosuppressive drug use, adjusted for demographic and medical factors, and the different outcomes as specified above. Variables were entered in multivariate regression and were forward: conditional selected. To determine the predictive accuracy for the different scores, we calculated sensitivity, specificity, area under the receiver operating curve (AUROC), and Brier scores. We created confidence intervals by bootstrapping 2000 samples. Differences in AUROCs between nested models were tested using the approach of Robin et al. [18].

## Results

### Patient characteristics

We included 1516 patients with a median age of 64 years, of which 42.5% were female (Table 1). Overall, 53.1% of the patients had a diagnosis of sepsis according to the Sepsis-3 criteria (SOFA or qSOFA ≥ 2) (Table 1). The population was subdivided by the use of immunosuppressives. In total 614 (40.5%) patients used one or more immunosuppressive drugs, of which glucocorticoids were the most used: the cohort consisted of 496 (32.7%) glucocorticoid users (Table S1, appendix). Of the glucocorticoid users, 237 (15.6%) used < 7.5 mg/day, 117 (7.7%) used 7.5–15 mg/day, and 142 (9.4%) used > 15 mg/day. Other immunosuppressives used were TNF blockers (1.3%), cellular immunosuppressives (12.0%), selective immunosuppressives (1.8%), IMPDH inhibitors (11.2%), purine antagonist (3.8%), and folic acid antagonist (2.4%) (Table S2, appendix). According to the Sepsis-3 criteria, sepsis was more prevalent among patients using 0–15 mg glucocorticoids per day (64.6–68.4%) and patients using IMPD inhibitors (61.8%) as compared to the whole cohort (53.1%, Table S2, appendix).

**Table 1:** Baseline characteristics

Variables	Total population (*n* = 1516)	Immunocompromised (*n* = 614, 40.5%)	Glucocorticoids (0–7.5 mg) (*n* = 237, 15.6%)	Glucocorticoids (7.5–15 mg) (*n* = 117, 7.7%)	Glucocorticoids (> 15 mg) (*n* = 142, 9.4%)
Age (years)	64 (51, 72)	62 (51, 69)*	60 (50, 68)*	65 (52, 71)	63 (53, 69)
Female	645 (42.5)	281 (45.8)*	119 (50.2)*	36 (30.8)*	61 (43.0)
Solid organ transplant	236 (15.6)	236 (38.6)*	155 (65.7)*	51 (43.6)*	9 (6.4)*
Bone marrow transplant	101 (6.7)	78 (11.4)*	8 (3.4)*	17 (14.5)*	21 (14.8)*
Malignancy with solid tumor	405 (27.0)	131 (21.4)*	34 (14.3)*	22 (19.0)	57 (40.1)*
Hematologic malignancy	300 (20.0)	134 (21.9)	19 (12.2)*	28 (24.1)	40 (28.2)*
Heart rate (bpm)	95 (80, 108)	95 (80, 108)	90 (75, 102)*	95 (82, 106)	98 (90, 110)*
MAP (mmHg)	93.3 (83.3, 102.1)	93.0 (83.3, 103.0)	94.7 (81.7, 103.3)	91.7 (82.0, 102.7)	92.7 (84.0, 102.1)
Respiratory rate (/min)	18 (16, 23)	18 (16, 22)	18 (16, 22)	20 (16, 23)	18 (16, 22)
Oxygen saturation (%)	97 (95, 98)	97 (95, 98)	97 (96, 98)*	96 (94, 98)	96 (93, 98)*
Body temperature (°C)	37.5 (36.8, 38.4)	37.5 (36.8, 38.3)	37.4 (36.7, 38.2)	37.8 (36.8, 38.6)	37.4 (36.7, 38.0)
CRP at ED (mg/L)	78 (34, 156)	70 (32, 135)*	75 (37, 136)	72 (24, 135)	75 (35, 147)
Leukocyte count at ED (10^9^/L)	9.5 (6.2, 13.9)	9.1 (5.7, 13.2)*	9.8 (7.3, 13.5)	9.1 (5.8, 14.20)	7.9 (3.3, 11.9)*
SOFA score at ED	2 (1, 3)	2 (1, 3)*	2 (1,3)*	2 (1, 4)*	2 (1, 3)
Sofa ≥ 2 at ED	908 (51.2)	361 (58.7)*	150 (63.3)*	**79 (67.5)**	70 (49.3)
qSOFA score at ED	0 (0, 1)	0 (0, 1)	0 (0,1)	0 (0, 1)	0 (0, 1)
SIRS score at ED	2 (1, 3)	2 (1, 3)	1 (1,3)*	2 (1, 3)	2 (1, 3)
NEWS score at ED	2 (1, 5)	2 (1, 5)	2 (1,4)*	3 (1,5)	3 (1, 5)
Sepsis (according to Sepsis-3)	805 (53.1)	363 (59.1)*	153 (64.6)*	80 (68.4)*	72 (50.7)
LOS	4 (1, 8)	4 (1, 8)	5 (2, 8)	5 (3, 8)	3 (0, 7)
ICU admission	77 (5.1)	30 (4.9)	9 (3.8)	8 (6.8)	10 (7.0)
In-hospital mortality	55 (3.6)	20 (3.3)	5 (2.1)	2 (1.7)	12 (8.5)*
28-day mortality	94 (6.2)	36 (5.9)	13 (4.6)	6 (5.1)	17 (12.0)*

Data are presented as number (percentage) for categorical variables and median (IQR [inter quartile range]) for continuous variables. *P*-values were calculated using a Mann–Whitney *U* test or Chi-squared test for categorical variables. A *p*-value of < 0.05 was considered significant and is indicated in bold. Solid organ transplant is a combination of renal, liver, and other solid organ transplant

Sepsis according to Sepsis-3 criteria based on combination of suspected/confirmed infection and (q)SOFA ≥ 2. Total population: including patients using immunosuppressives. Immunocompromised: patient using any kind of immunosuppressives

*CRP* C-reactive protein, *ED* emergency department, *SOFA* Sequential Organ Failure Assessment, *qSOFA* quick Sequential Organ Failure Assessment, *SIRS* Systemic Inflammatory Response Score, *NEWS* National Early Warning Score, *LOS* length of stay in hospital, *ICU* intensive care unit

*p < 0.05 as compared to all patients (excluding the test group)

### Outcomes of sepsis in patients on immunosuppressives

In total, 77 (5.1%) of the patients were admitted to the ICU, 55 (3.6%) died in hospital, and 94 (6.2%) died within 28 days after the ED visit. Both the in-hospital and 28-day mortality rates were higher for patients using high-dose (> 15 mg/day) glucocorticoids, being 8.5% and 12.0%, respectively, while the number of patients admitted to the ICU did not differ (Table 1). Univariate analysis demonstrated a threefold increased risk of in-hospital and 28-day mortality among high-dose glucocorticoid users (Tables 2 and S3). For low- and intermediate-dose glucocorticoids, the in-hospital mortality rate, 28-day mortality rate and ICU admission rate did not differ from the overall population (Table 1). Use of either low- or intermediated dose glucocorticoids was not associated with increased risk of ICU admission, in-hospital or 28-day mortality (Tables 2, S3, and S4). The in-hospital mortality rate, 28-day mortality rate and ICU admission rate did not differ between patients using other classes of immunosuppressive drugs, except an unexpected lower 28-day mortality among patients using IMPDH inhibitors (Table S2). Folic acid antagonist use was associated with an increased risk of ICU admission, but not in-hospital or 28-day mortality (Tables 2, S3, and S4). In summary, use of high-dose glucocorticoids (> 15 mg/day) was associated with increased risk of both in-hospital and 28-day mortality, use of IMPD inhibitors was associated with a lower 28-day mortality, and folic acid antagonist use was associated with an increased risk of ICU admission.

**Table 2:** In-hospital mortality from infection for subgroups of immunosuppressives

Variable	OR	95% CI	*p*-value
Any immunosuppressive	0.83	0.47–1.44	0.525
IMPDH inhibitors	0.29	0.04–0.94	0.088
Purine antagonist	0.00	< 0.01–1 × 10^3^	0.978
Folic acid antagonist	1.11	0.06–5.40	0.920
Selective immunosuppressive	0.98	0.05–4.75	0.987
TNF blocker	0.00	< 0.01–1 × 10^14^	0.981
Cellular immunosuppressive	0.67	0.28–1.41	0.336
Glucocorticoids < 7.5 mg	0.53	0.18–1.22	0.181
Glucocorticoids 7.5 – 15 mg	0.44	0.07–1.44	0.261
Glucocorticoids > 15 mg	2.86	1.41–5.39	**0.002**

Univariable logistic regression in patients at the ED with infection for no immunosuppressive versus use of a certain immunosuppressive subgroup

### Sepsis screening tools in patients using glucocorticoids

To further explore the predictive performance of qSOFA and NEWS in patients using glucocorticoids, we first investigated the difference in the scores at baseline. The SOFA score was higher in patients with low and intermediate dose of glucocorticoids compared to the rest of the cohort. The SIRS and NEWS are lower in patients using low-dose glucocorticoids as compared to the rest of the cohort. No differences in sepsis screening tools were found between patients using high-dose glucocorticoids and patients without high-dose glucocorticoids (Table 1). The sepsis scores of patients using other types of immunosuppressives are depicted in the supplemental data (Table S2).

#### qSOFA

All individual qSOFA score items and use of high-dose glucocorticoids were univariately associated with an increased risk of in-hospital mortality (Table 3). Multivariate regression analysis showed that use of high-dose glucocorticoids was independently associated with an increased risk of in-hospital mortality (OR 3.22 [95% CI 1.56–5.99], *p* < 0.05; Table 3). Other factors predictive of in-hospital mortality in this model were systolic blood pressure < 100 mmHg and respiratory rate ≥ 22/min, but not consciousness (EMV < 15).

**Table 3:** qSOFA as predictor of in-hospital mortality complemented with use of immunosuppressives

	Univariate OR (95%CI)	*p*-value	Multivariate OR (95% CI)	*p*-value	OR (95%CI)	*p*-value
Constant			**0.02 (0.01–0.03)**	**< 0.001**	**0.02 (0.01–0.02)**	**< 0.001**
qSOFA score items						
Systolic blood pressure ≤ 100 mm/Hg	**3.02 (1.52–5.61)**	**< 0.001**	**2.41 (1.19–4.57)**	**0.009**	**1.49 (1.22–6.22)**	**0.007**
Respiratory rate ≥ 22/min	**3.69 (2.14–6.49)**	**< 0.001**	**3.27 (1.87–5.80)**	**< 0.001**	**3.37 (1.92–6.00)**	**< 0.001**
EMV score < 15	**2.98 (1.33–6.01)**	**0.004**	1.89 (0.82–3.94)	0.110	1.96 (0.84–4.12)	0.092
Immunosuppressives						
Glucocorticoids < 7.5 mg	0.53 (0.18–1.22)	0.181	n.s			
Glucocorticoids 7.5–15 mg	0.44 (0.07–1.44)	0.261	n.s			
Glucocorticoids > 15 mg	**2.86 (1.41–5.39)**	**0.002**			**3.22 (1.56–5.99)**	**< 0.001**

Significant values are represented in bold, with the significant value in column *p*-value.

Univariate and multivariate regression analysis with in-hospital mortality as outcome. Data are presented as odds ratio (OR) (95% CI), level of significance *p* < 0.05. For univariable analysis, all *p* < 0.200 are entered into multivariate analysis

Model characteristics for qSOFA: *R* = 0.020, *df* = 3, *p* < 0.05, *N* = 1516. Model characteristics for qSOFA with high-dose glucocorticoids: *R* = 0.026, *df* = 4, *p* < 0.05, *N* = 1516

*n.s.* not significant in multivariate analysis, *qSOFA* quick sequential organ failure score, *EMV score* eye movement verbal score

#### NEWS

Abnormal NEWS scores for respiratory rate (< 12 or > 20/min), oxygen suppletion and saturation (SpO_2_ < 96%), systolic blood pressure (< 111 or > 219 mmHg), heart rate (< 51 or > 90 bpm), and consciousness (EMV < 15) were univariately associated with in-hospital mortality (Table 4). When adjusting the model for use of high-dose glucocorticoids, we identified a threefold increased risk for in-hospital mortality among high-dose glucocorticoid users (OR 3.03 [95% CI 1.45–5.91], *p* < 0.05; Table 4), while oxygen suppletion and abnormal respiratory rate and systolic blood pressure are other independent predictors from the NEWS.

**Table 4:** NEWS as predictor of in-hospital mortality, complemented with use of immunosuppressives

	Univariate OR (95%CI)	*p*-value	Multivariate OR (95%CI)	*p*-value
Constant			0.01 (0.01–0.02)	< 0.001
NEWS score items				
Respiratory rate < 12 or > 20	**1.66 (1.36–2.04)**	**< 0.001**	**1.44 (1.16–1.80)**	**0.001**
Oxygen saturation < 96%	**1.43 (1.11–1.82)**	**0.004**	n.s	
Oxygen suppletion	**2.31 (1.74–3.04)**	**< 0.001**	**1.78 (1.31–2.40)**	**< 0.001**
Temperature ≤ 36 or > 38 °C	0.85 (0.49–1.40)	0.540	n.s	
SBP < 111 or > 219 mmHg	**1.68 (1.28–2.16)**	**0.001**	**1.445 (1.08–1.90)**	**0.008**
Heart rate < 51 or > 90 bpm	**1.73 (1.23–2.45)**	**0.002**	n.s	
EMV < 15	**2.98 (1.33–6.01)**	**0.004**	n.s	
Immunosuppressives				
Glucocorticoids < 7.5 mg	0.53 (0.18–1.22)	0.181		
Glucocorticoids 7.5–15 mg	0.44 (0.07–1.44)	0.261		
Glucocorticoids > 15 mg	**2.86 (1.41–5.39)**	**< 0.002**	**3.03 (1.45–5.91)**	**0.002**

Significant values are represented in bold, with the significant value in column *p*-value

Univariate and multivariate regression analysis with in-hospital mortality. Data are presented as OR (95% CI), level of significance *p* < 0.05. For univariable analysis, all *p* < 0.200 are entered into multivariate analysis

Model characteristics for NEWS: *R* = 0.035, *df* = 7, *p* < 0.05, *N* = 1516. Model characteristics for NEWS with high-dose glucocorticoids: *R* = 0.036, *df* = 4, *p* < 0.05, *N* = 1516

*n.s.* not significant in multivariate analysis, *EMV score* eye movement verbal score, *SBP* systolic blood pressure

### Comparing the performance of qSOFA and NEWS in patients using high-dose glucocorticoids

**Fig. 2:**
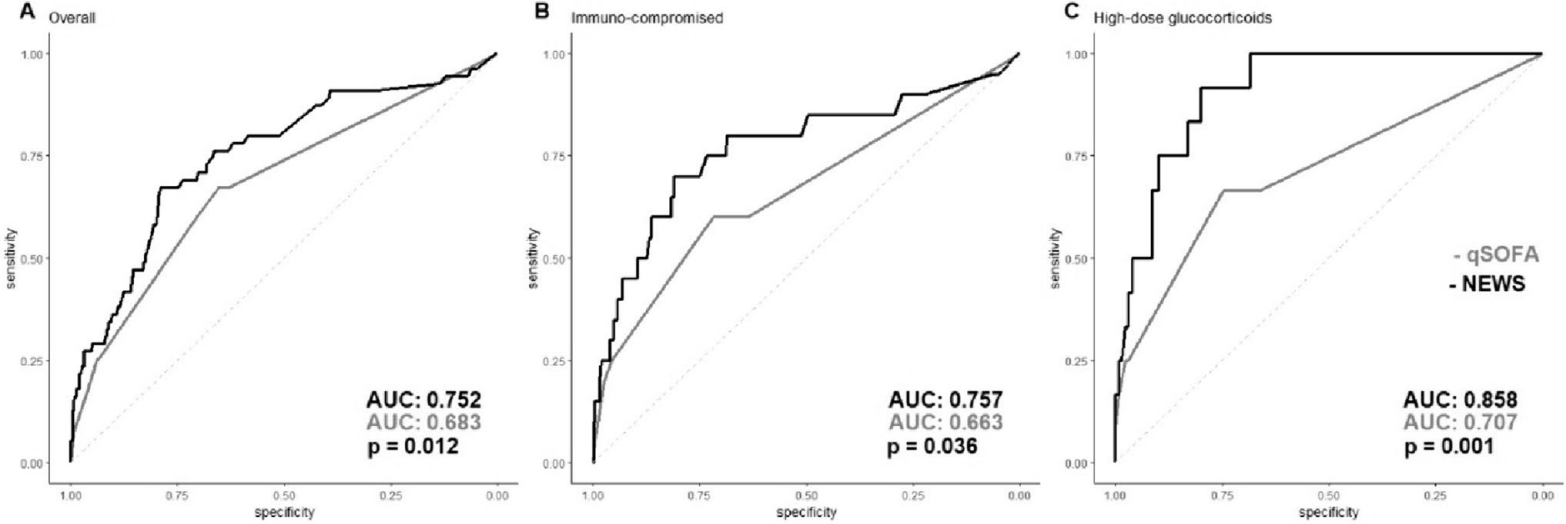
ROC curve of qSOFA and NEWS and in-hospital mortality. The AUC was determined using a bootstrap with 2000 steps. *qSOFA* quick Sequential Organ Failure Assessment, *NEWS* National Early Warning Score, *AUC* area under the curve. **A** ROC curve in the overall population where NEWS (AUC: 0.752) had a higher AUC compared to qSOFA (AUC: 0.683) *p* < 0.05. **B** ROC curve in the immunocompromised population where NEWS (AUC: 0.757) had a higher AUC compared to qSOFA (AUC: 0.663) *p* < 0.05. **C** ROC curve in the high-dose glucocorticoids population where NEWS (AUC: 0.858) had a higher AUC compared to qSOFA (AUC: 0.707) *p* < 0.05

The sensitivity of qSOFA to predict 28-day mortality in the whole cohort was 25.4% and 25.0% for the immunocompromised (any class) and high-dose glucocorticoid group (Table 5). The specificity was 93.3% in the whole cohort, 92.4% in immunocompetent patients, 94.8% in immunocompromised patients, and 96.1% among patients using high-dose glucocorticoids. The sensitivity of the NEWS ranged from 65.4 to 100.0%, while the specificity was from 73.0 to 74.0% (Table 5). The sensitivity of the NEWS is higher among immunocompromised patients and those using high-dose glucocorticoids, whereas the specificity remained unaffected. The sensitivity of the SOFA ranged from 85.7 to 100.0%, while the specificity was from 40.4 to 51.5% (Table 5). The AUROC of the NEWS (0.752) to predict in-hospital mortality was higher than the qSOFA (0.683, *p* < 0.05, Fig. 2) among the whole study population. Specifically, among immunocompromised patients, the AUROC of NEWS (0.757) and SOFA (0.800) scores were higher as compared to the qSOFA (0.663, *p* < 0.05, Table 5, Fig. 2). In patients using high-dose glucocorticoids, the NEWS (0.858) and SOFA (0.899) were better in predicting in-hospital mortality as compared to qSOFA (0.707, *p* < 0.05, Table 5, Fig. 2).

**Table 5:** Predictive accuracy of qSOFA, adjusted qSOFA, NEWS, for in-hospital mortality in total cohort, immunocompetent group, immunocompromised group, and group on high-dose glucocorticoid therapy

	Overall (1516)	Immunocompetent (902)	Immunocompromised (614)	High-dose glucocorticoids (142)
qSOFA score				
< 2	41 (2.9)	26 (3.2)	15 (2.6)	9 (6.7)
≥ 2	14 (12.5)	9 (11.5)	5 (14.7)	3 (37.5)
Predictive accuracy (%)				
Sensitivity	25.4	25.7	25.0	25.0
Specificity	93.3	92.4	94.8	96.1
AUROC	0.683 (0.611–0.758)	0.694 (0.617–0.786)	0.665 (0.552–0.795)	0.707 (0.575–0.865)
NEWS score				
< 5	19 (1.7)	15 (2.3)	4 (0.9)	0 (0.0)
≥ 5	36 (8.5)	20 (7.8)	16 (9.5)	12 (26.7)
Predictive accuracy (%)				
Sensitivity	65.4	57.1	80.0	100.0
Specificity	73.5	73.1	74.0	73.0
AUROC	0.752 (0.699–0.828)	0.750 (0.690–0.847)	0.757 (0.669–0.900)	0.858 (0.881–0.993)
SOFA score				
< 2	6 (0.9)	5 (1.1)	1 (0.4)	0 (0.0)
≥ 2	49 (5.9)	30 (6.6)	19 (5.1)	12 (16.0)
Predictive accuracy (%)				
Sensitivity	89.1	85.7	95.0	100.0
Specificity	46.7	50.9	40.4	51.5
AUROC	0.666 (0.660–0.835)	0.657 (0.521–0.776)	0.800 (0.809–0.951)	0.899 (0.854 -0.979)

Data are presented as number (percentage) or area under the curve (AUC) (95% CI)

Adjusted qSOFA and NEWS score: 1 point extra for use of high-dose glucocorticoids, qSOFA cut-off value ≥ 2; NEWS cut-off value ≥ 5; SOFA cut-off value ≥ 2. AUROC was determined using a bootstrap with 2000 steps

*qSOFA* quick Sequential Organ Failure Assessment, *NEWS* National Early Warning Score, *AUROC* Area Under the Receiver Operating Curve

## Discussion

Here, we evaluated the effect of immunosuppressive drugs on the performance of commonly used sepsis scores to predict clinically relevant outcomes of sepsis. Of all classes of immunosuppressives studied, only the use of high-dose glucocorticoids (> 15 mg/day) was associated with a profoundly increased risk of in-hospital mortality, which remained significant in multivariate analysis. When comparing the performance of qSOFA and NEWS in predicting in-hospital mortality and attempting to adjust for high-dose glucocorticoid use, we found that NEWS is better in predicting in-hospital mortality than the qSOFA among immunocompromised patients.

A common concern in patients on immunosuppressive agents is a blunted immune response leading to a lack of clinical symptoms that usually accompany sepsis [13]. Subsequently, early sepsis recognition can be more challenging in immunocompromised patients. In our cohort we found a sensitivity around 25% and specificity ranging from 92.4 to 96.1% for qSOFA to predict in-hospital mortality. The highest specificity of the qSOFA was found in patients on high-dose glucocorticoids. The sensitivity of qSOFA was slightly lower in our immunocompromised groups, being 25.0% versus 25.7% in immunocompetent patients. The qSOFA is known to suffer from a low sensitivity for predicting both in-hospital and 28-day mortality, ranging from 15 to 48% in other studies, while the reported specificity is around 90% and higher [13, 19]. The qSOFA has a sensitivity of 47.8% and specificity of 90.5% among sepsis patients who are immunocompromised secondary to hematopoietic cell transplantation [9, 13, 19–21]. The sensitivity we found is within the wide range as reported by others. However, it appears lower as compared to the sensitivity of the qSOFA among hematopoietic cell transplantation receivers—on the other hand, the specificity of the qSOFA in our cohort of pharmacologically immunocompromised groups was slightly higher. Although qSOFA is considered more user-friendly, since it requires only three, readily available parameters, clinicians should be aware that identification of immunocompromised patients at high risk of poor outcome is hampered.

In our cohort, NEWS outperformed the qSOFA in predicting in-hospital mortality, both among the whole cohort as well as the studied subgroups. The AUROC to predict in-hospital mortality of the NEWS score was higher than the qSOFA in the whole cohort, as well as among immunocompromised patients and among those using high-dose glucocorticoids. The sensitivity of the NEWS ranged from 65.4% in the whole cohort to 80% in immunocompromised patients and 100% among high-dose glucocorticoid users. The specificity ranged from 73.0 to 74.0%. These findings are in line with previously reported sensitivity of 74.0–87.9% and specificity of 42.1%–90.2% to predict sepsis-related mortality in an immunocompetent population has been reported [9, 10, 20]. When comparing our findings among pharmacologically immunocompromised patients to the accuracy of NEWS among other groups of immunocompromised patients due to hematopoietic cell transplantation, it appears that the sensitivity is higher (hematopoietic cell transplantation: 64.9%), while specificity is similar (hematopoietic cell transplantation: 71.2%) [13]. Further, in kidney transplant recipients the NEWS and SOFA performed best in recipients compared to immunocompetent patients, compared to CRB-65, CURB-65, DS-CRB-65, qSOFA, and PSI (Pneumonia Severity Index) score [22]. In our study, the predictive performance of NEWS was higher than qSOFA in the whole cohort, as well as in immunocompromised patients in general and specifically in patients on high-dose glucocorticoids, in line with observations among patients with a suspected infection after hematopoietic cell transplantation [13].

The 28-day mortality rates in our study were 6.2% for the total cohort and 5.9% among patients who were pharmacologically immunocompromised. It should be noted that the studied population represents a group of patients with a severe infection at risk of developing sepsis and those who already meet sepsis criteria: a clinically relevant group of patients seen daily at the ED. More than half of the patients met Sepsis-3 criteria, while 5.1% was admitted to the ICU and 3.6% died in hospital. The ICU admission rate appears to be lower than in other studies, which could be explained by the relatively low number of patients with sepsis. Another explanation might be the support for more complex care at the general wards by ICU outreach teams in the Netherlands, which lowers the requirement for ICU admission. For the different groups of immunosuppressives, 28-day mortality rates ranged from 0 to 12%, with the highest mortality rate observed in patients on high-dose (> 15 mg/day) glucocorticoids. Although infections are a well-known risk associated with use of immunosuppressive drugs, the risk of deterioration and sepsis development varies between different classes of immunosuppressive drugs due to their different target cell(s) [17, 23–26]. While some drugs have very specific targets, glucocorticoids have broad effects on the immune system by their anti-inflammatory properties [27]. Previous studies among immunocompromised patients with an infection demonstrated similar or even better outcomes as compared to immunocompetent patients [5, 12, 28–30], with 28-day mortality rates from 8 to 32% [5, 12, 31]. Only a few studies took the specific immunosuppressive drug class and/or dose administered into account, rather than considering a general immunocompromised state when using any immunosuppressive drug or having specific co-morbidity (e.g., cancer, history of solid organ transplantation). The relevance of investigating specific immunosuppressive drug classes is supported by our findings, demonstrating an increased in-hospital and 28-day mortality risk among high-dose glucocorticoid users, while use of IMPDH inhibitors was associated with a lower 28-day mortality risk. In a study among rheumatic patients on either conventional/biological disease-modifying anti-rheumatic drugs (cs/b DMARDs) or glucocorticoids who developed sepsis, high-dose glucocorticoids were also associated with mortality, while bDMARD users had a lower risk on mortality [32]. In contrast, however, a study using health care insurance data demonstrated lower in-hospital mortality from sepsis among glucocorticoid users (not stratified to the dose) and among patients taking non-steroid immunosuppressive drugs. Yet, patients taking immunosuppressive drugs were more frequently admitted to the ICU because of sepsis and had a longer length of stay in hospital [29]. Unfortunately, the drug class was not further specified among the non-steroid immunosuppressive drug users.

### Strengths and limitations

There were several limitations to our study. First, the diagnosis of sepsis was based upon the combination of a (suspected) infection and a qSOFA/SOFA score of ≥ two according to information present in the electronic patient chart. Patients in our dataset could well be using ≥ one immunosuppressive drug at the same time and interactions and effects of concomitantly used agents cannot be ruled out. We stratified patients into groups based on the immunosuppressive drug use to identify specific drugs that would affect the predictive performance of the qSOFA and NEWS. Consequently, a number of patients on TNF blocker and selective immunosuppressives are small, potentially underpowered, and therefore at risk for type II error. Although we do not have evidence to support that the inclusion time from 8:00 to 23:00 h may have caused selection bias, the risk of such bias should be taken into account when interpreting the evidence provided in the study. It should be noted that there is an inherent risk of overfitting the model that we developed in this single-center cohort. Although the results do allow to expand understanding of the potential risks of immunosuppressives among patients with an infection at the ED, the model should not be used to screen patients without external validation.

## Conclusion

We found NEWS to have better discriminative accuracy than qSOFA in predicting in-hospital mortality from sepsis. Patients using more than 15 mg glucocorticoids per day (prednisone equivalent dosage) are particularly at risk for in-hospital mortality. Adjusting the NEWS for high-dose glucocorticoid use does not further improve its predictive performance. As a clinical consequence, we recommend using the NEWS to identify patients with infection at high risk of poor outcome.

## Supplementary Information

Below is the link to the electronic supplementary material.

Supplementary file 1 (DOCX 27 KB)

## Data Availability

Data and script are available upon reasonable request.
