# Correction to: Predictive performance of NEWS and qSOFA in immunocompromised sepsis patients at the emergency department

**DOI:** 10.1007/s15010-024-02282-1

**Published:** 2024-04-26

**Authors:** Lisanne Boekhoud, Helena M. E. A. Schaap, Rick L. Huizinga, Tycho J. Olgers, Jan C. ter Maaten, Douwe F. Postma, Hjalmar R. Bouma

**Affiliations:** 1 Department of Clinical Pharmacy and Pharmacology, University Medical Center Groningen, University of Groningen, P.O. Box 30.001, EB70, 9700 RB Groningen, The Netherlands; 2 Department of Internal Medicine, University Medical Center Groningen, University of Groningen, Groningen, The Netherlands

**Correction to: Infection** 10.1007/s15010-024-02247-4

In this article on page 8, right column, line 13 the abbreviation of

PSI (Patient safety indicators) is wrong.

Correct is:

PSI (Pneumonia Severity Index).

The Original Article has been corrected.

