# Supplementary material for: Predictive performance of NEWS and qSOFA in immunocompromised sepsis patients at the emergency department
Source: Infection. 2024 Apr 12;52(5):1863–73. doi: 10.1007/s15010-024-02247-4 (PMC11499318; doi:10.1007/s15010-024-02247-4)
Supplement: Supplementary file 1 — (DOCX 27 KB) [file 15010_2024_2247_MOESM1_ESM.docx]

Supplemental tables

Table S1. Overview of groups of immunosuppressives

| **Group of immunosuppressive** | **Drugs** | **Total*** |
| --- | --- | --- |
| Cellular immunosuppressives | Tacrolimus (N = 187), Cyclosporine (N = 61), Sirolimus (N = 13), Everolimus (N = 20) | 267 |
| TNF-α blockers | Adalimumab (N = 8), Etanercept (N = 5), Infliximab (N = 6) | 19 |
| Selective immunosuppressives | Leflunomide (N = 3), Lenalomide (N = 17), Pomalidomide (N = 4), Thalidomide (N = 1), Anakinra (N = 2), Tocilizomab (N = 1) | 28 |
| Folic acid antagonist | Methotrexate (N = 37) | 37 |
| Purine antagonist | Azathioprine (N = 50), Mercaptopurine (N = 3), Tioguanine (N = 5) | 58 |
| IMPDH-inhibitors | Mycophenolate (N = 130), Mycophenlic acid (N = 73) | 170 |
| Glucocorticoids | Prednisolone (N = 402), Dexamethasone (N = 61), Hydrocortisone (N = 39), Betamethasone (N = 4), Cortison (N = 5) | 496 |

*If a patient uses >1 drug within a group of immunosuppressive, this is counted as 1. Abbreviations: TNF-α = tumor necrosis factor alpha; IMPD-inhibitor: Inosine-5’-monophosphate dehydrogenase inhibitor.

Table S2. Baseline characteristics

| **Variables** | **Total population**  (n=1516) | **Glucocorticoids**  **(0 – 7.5 mg)**  (n=237, 15.6%) | **Glucocorticoids**  **(7.5 – 15 mg)**  (n=117, 7.7%) | **Glucocorticoids**  **(> 15 mg)**  (n=142, 9.4%) | **TNF blocker**  (n=19, 1.3%) | **Cellular**  (n=267, 17.6%) | **Selective**  **(**n=28, 1.8%) | **IMDPH-inhibitor**  (n=170, 11.2%) | **Purine-antagonist**  (n=58, 3.8%) | **Folic acid antagonist**  (n=37, 2.4%) |
| --- | --- | --- | --- | --- | --- | --- | --- | --- | --- | --- |
| Age | 64 (51, 72) | **60 (50, 68)*** | 65 ( 52, 71) | 63 (53, 69) | **51 (39, 63)*** | **57 (46, 66)*** | 64 (59, 69) | **57 (48, 65)*** | **54 (33, 65)*** | **72 (65, 78)*** |
| Female | 645 (42.5) | **119 (50.2)*** | **36 (30.8)*** | 61 (43.0) | **17 (89.5)*** | 121 (45.3) | 14 (50.0) | 72 (42.4) | 30 (51.7) | 14 (37.8) |
| Ischemic heart disease | 173 (11.5) | 22 (9.3) | 15 (12.8) | 17 (12.0) | 0 (0.0) | **21 (7.9)*** | 2 (7.1) | 24 (14.1) | 6 (10.3) | 6 (16.2) |
| COPD | 140 (9.2) | 27 (11.4) | 8 (6.8) | 10 (7.0) | 0 (0.0) | 20 (7.5) | 3 (10.7) | 16 (9.4) | 5 (8.6) | 6 (16.2) |
| Connective tissue disease | 125 (8.3) | **31 (13.1)*** | 12 (10.3) | 13 (9.2) | **8 (42.1)*** | 17 (6.4) | **7 (25.0)*** | 10 (5.9) | 6 (10.3) | **15 (40.5)*** |
| Diabetes mellitus | 357 (23.8) | **69 (29.2)*** | 32 (27.4) | 28 (19.7) | 5 (26.3) | **89 (33.5)*** | 4 (14.3) | **62 (36.5)*** | **6 (10.3)*** | 6 (16.2) |
| Chronic kidney disease | 220 (14.7) | **95 (40.1)*** | **34 (29.1)*** | 13 (9.2) | 0 (0.0) | **111 (41.6)*** | 1 (3.6) | **87 (51.2)*** | 11 (19.0) | **0 (0.0)*** |
| Liver disease | 199 (13.1) | **51 (21.5)*** | 9 (7.7) | 16 (11.3) | 5 (26.3) | **57 (21.3)*** | 3 (10.7) | **34 (20.0)*** | **20 (34.5)*** | 2 (5.4) |
| SOT total | 236 (15.6) | **153 (65.7)*** | **51 (43.6)*** | 9 (6.4) | 0 (0.0) | **201 (75.3)*** | **0 (0.0)*** | **136 (80.0)*** | **22 (37.9)*** | **1 (2.7)*** |
| Renal transplant | 174 (11.6) | **121 (51.1)*** | **40 (34.2)** | **5 (3.5)*** | 0 (0.0) | **146 (54.7)*** | 0 (0.0) | **109 (64.1)*** | **15 (25.9)*** | 1 (2.7) |
| Liver transplant | 55 (3.7) | **29 (12.3)*** | 5 (4.3) | 5 (3.5) | 0 (0.0) | **43 (16.1)*** | 0 (0.0) | **24 (14.1)*** | **6 (10.3)*** | 0 (0.0) |
| Another organ transplant | 39 (2.6) | **18 (7.6)*** | **12 (10.3)*** | 0 (0.0) | 0 (0.0) | **32 (16.1)*** | 0 (0.0) | **17 (10.0)*** | 1 (1.7) | 1 (2.7) |
| Bone marrow transplant | 101 (6.7) | **8 (3.4)*** | **17 (14.5)*** | **21 (14.8)*** | 0 (0.0) | **41 (15.4)*** | **6 (33.3)*** | 8 (4.7) | 0 (0.0) | 3 (8.1) |
| Malignancy with solid tumor | 405 (27.0) | **34 (14.3)*** | 22 (19.0) | **57 (40.1)*** | **0 (0.0)*** | **28 (10.5)*** | 5 (17.9) | **11 (6.5)*** | **2 (3.4)*** | **4 (11.1)** |
| Hematologic malignancy | 300 (20.0) | **19 (12.2)*** | 28 (24.1) | **40 (28.2)*** | **0 (0.0)*** | 60 (22.5) | **16 (57.1)*** | 24 (14.1) | 10 (17.2) | 5 (13.5) |
| Heart rate (bpm) | 95 (80, 108) | **90 (75, 102)*** | 95 (82, 106) | **98 (90, 110)*** | 103 (93, 110) | 93 (80, 105) | 98 (87, 116) | **91 (78, 105)*** | 94 (79, 110) | 85 (75, 104) |
| MAP (mmHg) | 93.3 (83.3, 102.1) | 94.7 (81.7, 103.3) | 91.7 (82.0, 102.7) | 92.7 (84.0, 102.1) | 91.0 (86.0, 100.0) | **95.3 (86.5, 103.3)*** | 93.8 (83.3, 100.1) | 92.7 (85.6, 101.7) | 89.8 (82.0, 96.7) | 89.7 (81.7, 96.7) |
| Respiratory rate (resp/min) | 18 (16, 23) | 18 (16, 22) | 20 (16, 23) | 18 (16, 22) | 20 (16, 24) | 18 (15, 22) | 18 (16, 21) | 18 (16, 22) | 18 (15, 23) | 20 (16, 25) |
| Oxygen saturation (%) | 97 (95, 98) | 97 (96, 98)* | 96 (94, 98) | 96 (93, 98)* | 97 (95, 99) | **97 (96, 99)*** | 96 (93, 98) | **97 (96, 99)*** | **98 (96, 99)*** | **95 (94, 98)*** |
| Body temperature (˚C) | 37.5 (36.8, 38.4) | 37.4 (36.7, 38.2) | 37.8 (36.8, 38.6) | 37.4 (36.7, 38.0) | 37.2 (36.8, 37.9) | 37.6 (36.7, 38.3) | 37.6 (36.8, 38.1) | 37.6 (36.7, 38.2) | 37.8 (37.0, 38.6) | 37.1 (36.7, 38.1) |
| CRP at ED (mg/L) | 78 (34, 156) | 75 (37, 136) | 72 (24, 135) | 75 (35, 147) | 61 (17, 89) | **64 (24, 116)*** | 70 (45, 146) | 74 (30, 120) | 70 (35, 116) | 78 (33, 150) |
| Leukocyte count at ED (10^9^/L) | 9.5 (6.2, 13.9) | 9.8 (7.3, 13.5) | 9.1 (5.8, 14.20) | **7.9 (3.3, 11.9)*** | 9.6 (5.8, 11.2) | 9.7 (6.5, 13.4) | **4.1 (2.2, 8.1)*** | **10.5 (7.4, 14.2)*** | **7.8 (5.5, 9.6)** | 8.8 (5.1, 14.0) |
| SOFA score at ED | 2 (1, 3) | **2 (1,3)*** | **2 (1, 4)*** | 2 (1, 3) | 1 (0, 3) | **2 (1, 3)*** | 2 (1, 3) | **2 (1, 3)*** | 2 (0, 3) | 2 (1, 3) |
| qSOFA score at ED | 0 (0, 1) | 0 (0,1) | 0 (0, 1) | 0 (0, 1) | 0 (0, 1) | **0 (0, 1)*** | 0 (0, 1) | 0 (0, 1) | 0 (0, 1) | 1 (0, 1) |
| SIRS score at ED | 2 (1, 3) | **1 (1,3)*** | 2 (1, 3) | 2 (1, 3) | 2 (1, 3) | 2 (1, 3) | 2 (2, 3) | 1 (1, 2) | 2 (1, 3) | 2 (1, 2) |
| NEWS score at ED | 2 (1, 5) | **2 (1,4)*** | 3 (1,5) | 3 (1, 5) | 3 (2, 5) | **2 (1, 4)*** | 3 (2, 5) | **2 (1, 4)*** | 3 (1, 4) | 4 (2, 5) |
| Sepsis (according to Sepsis-3) | 805 (53.1) | **153 (64.6)*** | **80 (68.4)*** | 72 (50.7) | 7 (36.8) | 168 (62.9)* | 17 (60.7) | **105 (61.8)*** | 31 (53.4) | 20 (54.1) |
| LOS | 4 (1, 8) | 5 (2, 8) | 5 (3, 8) | 3 (0, 7) | **3 (0, 4)*** | 4 (1, 8) | **2 (0, 4)*** | 5 (2, 8) | 5 (1, 10) | 6 (1, 10) |
| ICU admission | 77 (5.1) | 9 (3.8) | 8 (6.8) | 10 (7.0) | 0 (0.0) | 13 (4.9) | 1 (3.6) | 8 (4.7) | 2 (3.4) | 5 (13.5) |
| In hospital mortality | 55 (3.6) | 5 (2.1) | 2 (1.7) | **12 (8.5)*** | 0 (0.0) | 7 (2.6) | 1 (3.6) | 2 (1.2) | 0 (0.0) | 2 (5.4) |
| 28-day mortality | 94 (6.2) | 13 (4.6) | 6 (5.1) | **17 (12.0)*** | 0 (0.0) | 12 (4.5) | 1 (3.6) | **3 (1.8)*** | 1 (1.7) | 3 (8.1) |

Data are presented as number (percentage) for categorical variables and median (IQR [inter quartile range]) for continuous variables. P values were calculated using a Mann Whitney U test or Chi-squared test for categorical variables. A p-value of <0.05 was considered significant and are bold with *­. Solid organ transplant is a combination of renal, liver and other solid organ transplant. Abbreviations: COPD = chronic obstructive pulmonary disease; CRP = C-reactive protein; ED = emergency department; SOFA = Sequential Organ Failure Assessment; qSOFA = quick Sequential Organ Failure Assessment; SIRS = Systemic Inflammatory Response Score; NEWS = National Early Warning Score; LOS = Length of stay in hospital; ICU = Intensive care unit. Sepsis according to Sepsis 3 criteria based on combination of suspected/confirmed infection and (q)SOFA ≥ 2. Total population: including patients using immunosuppressives. * means *p* < 0.05 as compared to all patients (excluding the test group).

Table S3. Univariate regression model for 28-day mortality from infection for subgroups of immunosuppressives

| **Variable** | **OR** | **95% CI** | **p-value** |
| --- | --- | --- | --- |
| Any immunosuppressive | 0.906 | 0.585 – 1.385 | 0.653 |
| IMPDH-inhibitors | 0.247 | 0.060 – 0.670 | **0.018*** |
| Purine-antagonist | 0.262 | 0.085 – 5.285 | 0.181 |
| Folic acid antagonist | 1.346 | 0.319 – 3.836 | 0.627 |
| Selective immunosuppressive | 0.555 | 0.030 – 2.653 | 0.566 |
| TNF-blocker | <0.001 | <0.01 – 1*10^14^ | 0.980 |
| Cellular immunosuppressive | 0.670 | 0.343 – 1.199 | 0.206 |
| Glucocorticoids < 7.5 mg | 0.701 | 0.348 – 1.281 | 0.281 |
| Glucocorticoids 7.5 – 15 mg | 0.805 | 0.308 – 1.737 | 0.617 |
| Glucocorticoids > 15 mg | 2.290 | 1.276 – 3.907 | **0.003** |

Univariable logistic regression in patients at the ED with infection for no immunosuppressive versus use of certain immunosuppressive subgroup. Level of significance p ≤ 0.05.

Table S4. Univariate regression model for ICU admission from infection for subgroups of immunosuppressives

| **Variable** | **OR** | **95% CI** | **p-value** |
| --- | --- | --- | --- |
| Any immunosuppressive | 0.602 | 0.602 – 1.557 | 0.778 |
| IMPDH-inhibitors | 0.913 | 0.339 – 2.263 | 0.814 |
| Purine-antagonist | 0.109 | 0.109 – 2.211 | 0.584 |
| Folic acid antagonist | 3.053 | 1.021 – 7.425 | **0.019*** |
| Selective immunosuppressive | 1.101 | 0.061 – 5.468 | 0.715 |
| TNF-blocker | <0.001 | <0.01 – 1*10^14^ | 0.984 |
| Cellular immunosuppressive | 1.094 | 0.523 – 2.082 | 0.863 |
| Glucocorticoids < 7.5 mg | 0.528 | 0.219 – 1.086 | 0.330 |
| Glucocorticoids 7.5 – 15 mg | 1.198 | 0.521 – 2.404 | 0.396 |
| Glucocorticoids > 15 mg | 1.859 | 0.935 – 3.411 | 0.266 |

Univariable logistic regression in patients at the ED with infection for no immunosuppressive versus use of certain immunosuppressive subgroup. Level of significance p ≤ 0.05.
